# Mg/Si- and Ag-Doped Carbon-Based Media Rainwater Filtration System for Multiple Pollutants Removal

**DOI:** 10.3390/ma17225638

**Published:** 2024-11-18

**Authors:** So Yeon Yoon, Hyeseong Kim, Reneesha Valiyaveettil Basheer, Nurhaslina Abd Rahman, Seok Byum Jang, Kien Tiek Wong, Deok Hyun Moon, Choe Earn Choong, Min Jang

**Affiliations:** 1 Department of Environmental Engineering, Kwangwoon University, Seoul 01897, Republic of Korea; thdus997@naver.com (S.Y.Y.); cometk94@kw.ac.kr (H.K.); reneeshavb@gmail.com (R.V.B.); linarahman789@gmail.com (N.A.R.); seogbeom7088@naver.com (S.B.J.); kientiek@gmail.com (K.T.W.); 2 Plasma Bioscience Research Center, Kwangwoon University, Seoul 01897, Republic of Korea; 3 Department of Environmental Engineering, Chosun University, Gwangju 61452, Republic of Korea; dhmoon@chosun.ac.kr

**Keywords:** adsorption, palm shell activated carbon, rainwater, multi-pollutants, filtration

## Abstract

In this study, the removal performances of a multi-pollutant elimination cartridge system (MPECS) composed of palm shell waste-based activated carbon (PSAC), magnesium (Mg)/silicon (Si)-doped PSAC (Mg/Si-PSAC), and silver (Ag)-doped PSAC (Ag-PSAC) for heavy metals, organic pollutants, and *Escherichia coli* were investigated. Mg/Si impregnation significantly improved heavy metal removal using PSAC by increasing the surface area and adding more sorption sites to the magnesium silicate nanolayer. Fixed-bed column experiments showed that the MPECS column outperformed PSAC and commercial activated carbon (DJAC), with a 1.5 to 2.0 times higher *E. coli* removal and a higher removal of organic pollutants and heavy metals. The MPECS column, with its disinfection ability and adsorption of heavy metals and organic matter, is a promising system for removing multiple pollutants from rainwater.

## 1. Introduction

Water is essential for life and is vital to human health, ecosystems, and industrial and economic activities. However, water depletion is predicted owing to climate change and urbanization, with rising costs as supply struggles to meet demand [1]. South Korea ranks 30th out of 32 Organization for Economic Co-operation and Development (OECD) countries in terms of per capita water resources, with a utilization rate of 26.8%, which is far above the OECD average of 13% [2]. Rainwater can be a potential and usable water resource to secure more water resources for drinking and other applications. However, using untreated rainwater for drinking can harm health owing to contaminants such as bacteria, viruses, and micropollutants [3]. The harvested rainwater can be contaminated with microorganisms and heavy metals such as Al(III), Cd(II), or Cu(II) due to contact with dust particles or metal leaching from metal roofing materials or pipes [4]. Moreover, small-scale rainwater-harvesting systems often lack disinfection, which enables microorganism growth in storage tanks [5]. Therefore, effective pretreatment technology is crucial for removing micropollutants and microorganisms and preventing rainwater recontamination.

Various technologies such as coagulation, chemical oxidation, ion exchange, reverse osmosis, membrane separation, adsorption, and precipitation have been developed to treat rainwater by removing heavy metals and organic micropollutants [6]. However, coagulation, precipitation, and chemical oxidation require expensive or toxic chemicals, and they produce waste sludge [3]. Ion exchange and membrane separation also have limitations in rural areas because of their high maintenance and operational costs [7]. Among the various available technologies, adsorption is ideal for removing multiple contaminants owing to its low cost, high efficiency, and ease of operation and maintenance [8]. Low-cost adsorbents, such as activated carbon, biochar, and bentonite, are widely used in water purification because of their high surface area and functional groups [9]. Activated carbon is widely used for the treatment of micropollutants because of its availability, large surface area, and microporous structure [10]. In particular, activated carbon has nonselective sorption sites, with surface functional groups such as oxygen and hydrogen. Hydroxyl groups on activated carbon adsorb organic pollutants through hydrogen bonds and show a high affinity for their removal [11]. However, activated carbon has a poor sorption capacity and selectivity owing to its nonselective nature. Accordingly, surface modifications are required to improve its selectivity for inorganic compounds and microorganisms, allowing the simultaneous removal of various contaminants.

Our previous studies have shown that low-cost activated carbon from palm shell waste has a large surface area and high adsorption capacity for heavy metal or organic pollutant removal [12]. For example, modified palm shell waste-based activated carbon (PSAC) such as magnesium silicate-impregnated PSAC and magnetite-loaded PSAC exhibited high adsorption performances for fluoride and methylene blue removal [13,14]. Palm shell waste, which is abundant in Southeast Asia, especially in Malaysia, Indonesia, and Thailand, is a widely available source of biomass [15]. With a larger surface area and higher adsorption capacity than those of commercial adsorbents, PSAC is an economical and eco-friendly solution for removing multiple contaminants from rainwater. However, there are no studies on not only developing PSAC as a filter medium for the simultaneous removal of multiple contaminants in rainwater but also investigating its removal mechanisms.

In this study, we prepared surface-modified PSAC by impregnating it with magnesium/silicon (Mg/Si-PSAC) and silver (Ag-PSAC) for the selective adsorption of heavy metals (Cd, Cr, and Pb) and *E. coli* disinfection for the first time. The surface morphologies and functional groups of PSAC, Mg/Si-PSAC, and Ag-PSAC were analyzed by field emission scanning electron microscopy (FESEM), X-ray diffraction (XRD), and attenuated total reflectance (ATR)–Fourier-transform infrared (ATR-FTIR) spectroscopy. The heavy metal removal performances of PSAC, Mg/Si-PSAC, and Ag-PSAC were investigated using single or multiple heavy metals, with or without *E. coli*. Batch experiments showed that Mg/Si-PSAC and Ag-PSAC improved heavy metal adsorption compared with PSAC. The combinations of PSAC, Mg/Si-PSAC, and Ag-PSAC demonstrated superior heavy metal removal, even in the presence of *E. coli*. Based on the batch results, a multi-pollutant elimination cartridge system (MPECS) with four carbon layers (PSAC, Mg/Si-PSAC, Ag-PSAC, and PSAC) was developed for the effective removal of COD, heavy metals, and *E. coli* in fixed-bed column experiments. The MPECS column also showed superior *E. coli* disinfection and organic pollutant removal compared to the control columns with PSAC and commercial activated carbon (DJAC). The MPECS column containing Ag nanoparticles on Ag-PSAC inhibited microorganism growth up to 31,500 BV, meeting drinking water guidelines (N.D.). The MPECS column featuring Mg/Si-PSAC with a higher surface area and more active sites outperformed the control column in heavy metal removal. The C-O-Si-O-Mg-OH surface group in Mg/Si-PSAC selectively removed all heavy metals via adsorption and ion exchange, making MPECS a promising solution for treating multiple pollutants in rainwater.

## 2. Materials and Methods

### 2.1. Preparation of MPECS Filter Media

Details regarding the PSAC and its chemicals are available in the Supporting Information (SI) S1. Mg/Si was impregnated on PSAC by a hydrothermal method according to our previous published paper [13]. The PSAC was initially washed three to five times with distilled water to remove impurities. First, 3.3 g MgO and 4.8 g of SiO_2_ was dissolved in ultrapure water (50 mL). The suspension was stirred until a white magnesium silicate gel formed. Subsequently, 20 g of PSAC was added into the suspension and stirred at 150 rpm at 24 ± 1 °C for 1 h. Then, the mixtures were transferred to a Teflon-lined stainless steel autoclave, and then the autoclave was placed in an oven and heated at 150 °C for 10 h. The obtained Mg/Si-PSAC (Figure S1a) was filtered with 0.45 µm pore Whatman filter paper and washed 3 times with distilled water, and then oven-dried at 60 °C for 24 h.

The Ag-PSAC was prepared in the dark to prevent the photodecomposition of AgNO_3_ [16]. First, 10 g of PSAC was added to an AgNO_3_ solution, which was prepared by dissolving 0.158 g of AgNO_3_ in 100 mL of ultrapure water. After 24 h of impregnation, the PSAC was washed five times with distilled water to remove excess AgNO_3_ that was loosely adsorbed on the PSAC surface, until no silver-colored solids were observed in the filtrate. By adding 100 mL of 0.2 M NaBH_4_, the impregnated AgNO_3_ was chemically reduced (over 24 h) to form Ag particles (Ag^0^). After that, the obtained Ag-PSAC (Figure S1b) was washed with distilled water several times to remove excess NaBH_4_ and then dried at 24 ± 1 °C.

### 2.2. Batch Experiment Setup

Batch experiments compared the heavy metal removal efficiencies of PSAC, Mg/Si-PSAC, and Ag-PSAC, and examined the impact of media combinations on performance. Before starting the batch experiments, all glassware was washed with ultrapure water. The kinetic experiments were conducted using 200 mL of single or mixed heavy metal solution (1 mg L^−1^) with 0.1 g of adsorbent. Experiments were conducted with each adsorbent under the following conditions: (1) single heavy metals [each 1 mg L^−1^ of Cd(II), Cr(VI), or Pb(II)], (2) mixed heavy metals [1 mg L^−1^ of Cd(II), Cr(VI), and Pb(II) together], and (3) mixed heavy metals with *E. coli* (1.0 × 10^4^ CFU 100 mL^−1^). After evaluating their removal efficiencies, four combinations of carbon media were tested for the simultaneous removal of mixed heavy metals and *E. coli*: (1) PSAC + Mg/Si-PSAC, (2) PSAC + Ag-PSAC, (3) Mg/Si-PSAC + Ag-PSAC, and (4) PSAC + Mg/Si-PSAC + Ag-PSAC. The experiments used an equal mass ratio (1:1) for two media (0.5 g each) and 1:1:1 for three media (each 0.33 g each). All batch tests were conducted with the suspensions agitated at 200 rpm at room temperature (25 ± 0.5 °C). The initial pH of the solution was not adjusted, because the solution pH ranged from to 6 to 7. Suspensions were collected at 5–60 min intervals, filtered through 0.45 μm PTFE syringe filters (Hyundai Micro Co., Ltd., Seoul, Republic of Korea), and analyzed for heavy metal and *E. coli* concentrations. The single heavy metal kinetic adsorption data were fitted to pseudo-first- and pseudo-second-order kinetic models to investigate the adsorption behavior of each PSAC (details are described in SI-S2). The Cd(II), Cr(VI), and Pb(II) concentrations were measured using an inductively coupled plasma optical emission spectrometer (ICP-OES, Perkin Elmer, Inc., AVIO 200, Waltham, MA, USA, minimum detection limits for Cd(II), Cr(VI), and Pb(II): 0.1, 0.2, and 1 µg L^−1^, respectively). The *E. coli* population in the water samples was determined using the plate counting method.

### 2.3. Column Experiments

Continuous fixed-bed column experiments were conducted to assess the practical applicability of the MPECS filter in removing heavy metals, organic pollutants, and microorganisms from rainwater. Synthesized rainwater was prepared and used in column experiments to investigate the breakthrough points and lifespan of the MPECS column. The 2 mL acryl columns were packed with four layers of metal-modified PSAC (PSAC, Mg/Si-PSAC, Ag-PSAC, and PSAC volume percentages of 27.8%, 22.2%, 22.2%, and 27.8%, respectively) (Figure S2). Based on the previous batch results, the MPECS filter was designed using a combination of PSAC, Mg/Si-PSAC, and Ag-PSAC, featuring four layers to remove heavy metals, organic pollutants, and *E. coli*. The first layer (P-1) used PSAC as a preliminary treatment to remove organic compounds. The second layer (P-2) used Mg/Si-PSAC, primarily for heavy metal removal. The third layer (P-3) was composed of Ag-PSAC acted as a disinfectant. The fourth layer (P-4) used PSAC to capture the remaining pollutants that were not removed by the previous layers. However, to treat the organic pollutants in rainwater, the MPECS filter used a higher volume of PSAC. Commercial granular activated carbon (DJAC) and pure PSAC were used as the control columns to evaluate the potential of the MPECS. Higher initial heavy metal concentrations [Al(III): 0.221–0.354 mg L^−1^, Cd(II): 0.029–0.121 mg L^−1^, Cr(VI): 0.073–0.087 mg L^−1^, Pb(II): 0.054–0.34 mg L^−1^] than those in actual rainwater (Table 1) were used to assess the breakthrough and endpoints of the carbon media [17,18].

The column experiments used an upward flow with a peristaltic pump at a constant rate of 4 mL min^−1^. The control columns were filled with unmodified PSAC or commercial granular activated carbon (DJAC, Daejung Chemicals and Metals Co., Ltd., Siheung, Republic of Korea). The detailed configurations of the column experiments are shown in Table S1. The effluents were sampled in 2 mL conical tubes at 50–5000 BV intervals. All the collected samples were filtered using a 0.45 µm Hyundai Micro syringe filter. The Al(III), Cd(II), Cr(VI), and Pb(II) concentrations were measured using ICP-OES. The *E. coli* population in the effluents was also determined using the pour plate method. Chemical oxygen demand (COD) was measured by the colorimetric method (USEPA method 410.3), using potassium dichromate (K_2_Cr_2_O_7_) to assess the organic pollutant concentrations in the effluents. The solution pH was measured using a multi-meter (HI 98104; Hanna Instruments, Smithfield, RI, USA).

### 2.4. Characterization

The surface morphologies of PSAC, Mg/Si-PSAC, and Ag-PSAC were analyzed using field emission scanning electron microscopy (FESEM)-energy dispersive spectrometry (FESEM-EDS, Jeol SEM, JSM-7001F, JEOL Ltd., Tokyo, Japan) at 15 kV. The specific surface area, pore volume, and pore diameter of PSAC, Mg/Si-PSAC, and Ag-PSAC were analyzed using Brunauer–Emmett–Teller (BET) and N_2_ adsorption–desorption isotherms (Micromeritics’ 3FLEX at −196 °C) after degassing at 110 °C for four hours. The XRD analysis was conducted utilizing a multi-purpose X-ray diffractometer (X’Pert Pro, PANalytical, KBSI, Seoul center) using Cu-Kα radiation (λ = 1.541 Å at 40 kV), with a scan angle in the range of 10–90°. The ATR-FTIR spectra were obtained to investigate the surface functional groups of the MPECS media using a Fourier-transform infrared spectrophotometer (IRSpirit, Shimadzu Scientific Korea, QATR-S, Seoul, Republic of Korea) over a wavelength range of 4000–399 cm^−1^.

## 3. Results

### 3.1. Heavy Metal Removal Using Carbon Media

**Figure 1:**
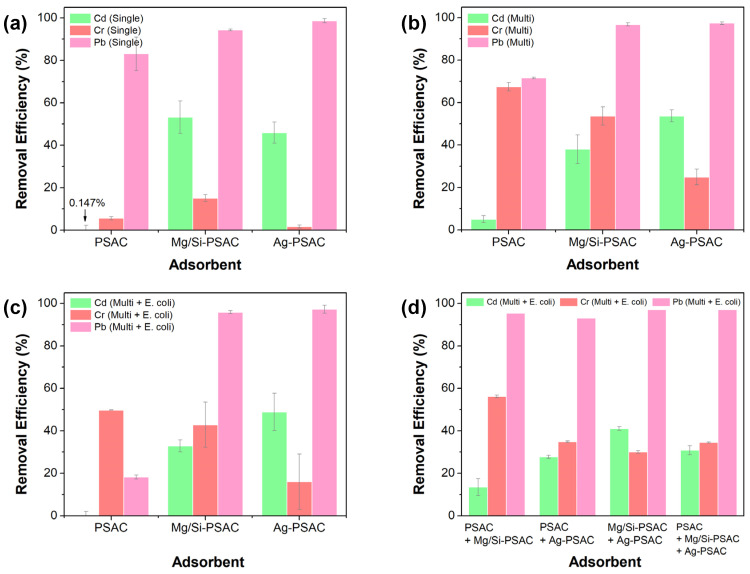
Heavy metal [Cd(II), Cr(VI), Pb(II)] removal efficiencies of PSAC, Mg/Si-PSAC, and Ag-PSAC in the presence of (**a**) single heavy metal, (**b**) multi-heavy metal, and (**c**) multi-heavy metal with microorganisms (*Escherichia coli*), and (**d**) heavy metal removal efficiencies of different carbon media combinations in the presence of multi-heavy metal and microorganisms (Combination 1—PSAC + Mg/Si-PSAC, 2—PSAC + Ag-PSAC, 3—Mg/Si-PSAC + Ag-PSAC, 4—PSAC + Mg/Si-PSAC + Ag-PSAC) (initial heavy metal concentration: each 1 mg L^−1^, initial E-coli population: 1.0 × 10^4^ CFU 100 mL^−1^, dosage: 0.1 g, solution volume: 200 mL, reaction time: 60 min, RT).

Before the column experiments, batch tests were performed to evaluate the heavy metal removal performance of each carbon-based medium. Batch experiments with PSAC, Mg/Si-PSAC, and Ag-PSAC were used to compare the Cd(II), Cr(VI), and Pb(II) removal efficiencies under single and mixed conditions (Figure 1a,b). Mg/Si-PSAC demonstrated a superior single-metal removal performance compared to PSAC and Ag-PSAC (Figure 1a and Figure S3, Table S2). PSAC showed the lowest Cd(II) adsorption (0.15%) and the lowest heavy metal removal efficiency among the tested PSACs. Ag-PSAC exhibited the highest Pb(II) removal efficiency (98.7%) but the lowest Cr(VI) removal efficiency (1.7%). Overall, Mg/Si and Ag impregnation improved the adsorption of Cd(II) and Pb(II) onto Mg/Si-PSAC, whereas Ag was less favorable for Cr(VI) adsorption. Cr(VI) is dominantly present as negative charged (CrO_4_^2−^) in a neutral pH, repelling the negatively charged PSAC, leading to a low adsorption efficiency [20]. In contrast, the positive charged silver nanoparticles doped on the surface of PSAC exhibited a better adsorption affinity towards negatively charged Cr(VI) compared to PSAC.

The heavy metal adsorption results under single conditions were fitted to pseudo-first- and pseudo-second-order kinetic models (Table S3). The R^2^ values obtained from the kinetic models indicated that the overall heavy metal adsorption on PSAC, Mg/Si-PSAC, and Ag-PSAC fitted well with the pseudo-second-order model, indicating that chemisorption was dominant for heavy metal removal [21].

In the presence of Cd(II), Cr(VI), and Pb(II), the Cr(VI) removal efficiency increased significantly in all carbon media (Figure 1b and Figure S4; Table S4). This is due to the co-precipitation of metals occurring on the surface of the PSACs causing the additional adsorption of Cr(VI) [22]. Hence, the co-precipitation of metals on carbon media surfaces enhances negatively charged Cr(VI) (CrO_4_^2−^) adsorption [22]. The PSAC showed a slight decrease in Pb(II) removal efficiency under multi-heavy metal conditions and, despite improved Cr(VI) removal (67.5%), a decrease in Cd(II) removal efficiency (5.1%). Mg/Si-PSAC and Ag-PSAC exhibited significantly improved Cr(VI) removal efficiencies (53.7% and 25.0%, respectively) and maintained higher Cd(II) removal rates (38.1% and 53.7%, respectively) than those of PSAC. This revealed that co-precipitating metals on surface-modified PSACs significantly improved their Cr(VI) removal efficiencies. For Pb(II) removal, Mg/Si-PSAC and Ag-PSAC still showed excellent removal performances at 60 min (96.8% and 97.4%, respectively).

To simulate microorganisms in rainwater, the effects of microorganisms on mixed heavy metal removal using the three different media were tested (Figure 1c and Figure S5, Table S5). The heavy metal removal performance of all the adsorbents decreased in the presence of *E. coli*. For the PSAC, the removal efficiencies of Cd(II), Cr(VI), and Pb(II) decreased significantly from 5.1 to 0%, 67.5 to 49.8%, and 71.6 to 18.2%, respectively. This indicates that PSAC’s adsorption capability is significantly hindered by microorganisms. In contrast, the heavy metal adsorption performances of Mg/Si-PSAC and Ag-PSAC were less affected by the presence of microorganisms than that of PSAC. The presence of *E. coli* altered the removal efficiencies of Mg/Si-PSAC and Ag-PSAC for Cd(II), Cr(VI), and Pb(II) from 38.1/53.7% to 32.9/48.9%; 53.7/25.0% to 42.9/16.0%; and 96.8 to 97.4% to 95.9 and 97.3%, respectively. This indicated that *E. coli* had a minimal interference with heavy metal adsorption on Mg/Si-PSAC and Ag-PSAC.

Moreover, the heavy metal removal efficiencies of various carbon media combinations were investigated (Figure 1d and Figure S6, Table S6). Four carbon media combinations were tested for the simultaneous removal of Cd(II), Cr(VI), and Pb(II). All combinations showed strong Pb(II) adsorption performances (≥93%). Combination 1 (PSAC + Mg/Si-PSAC) showed the highest Cr(VI) removal (56.2%) and lowest Cd(II) removal (13.5%). Combinations 2–4, containing Ag-PSAC, exhibited better Cd(II) removal than Combination 1. These results indicate that the Mg/Si or Ag impregnation of PSAC enhanced heavy metal adsorption. Based on these batch tests, heavy metals and E. coli were eliminated, and to conduct a long-term rainwater treatment, we established three different PSAC media: PSAC, Mg/Si-PSAC, and Ag-PSAC.

### 3.2. Material Characterization

**Figure 2:**
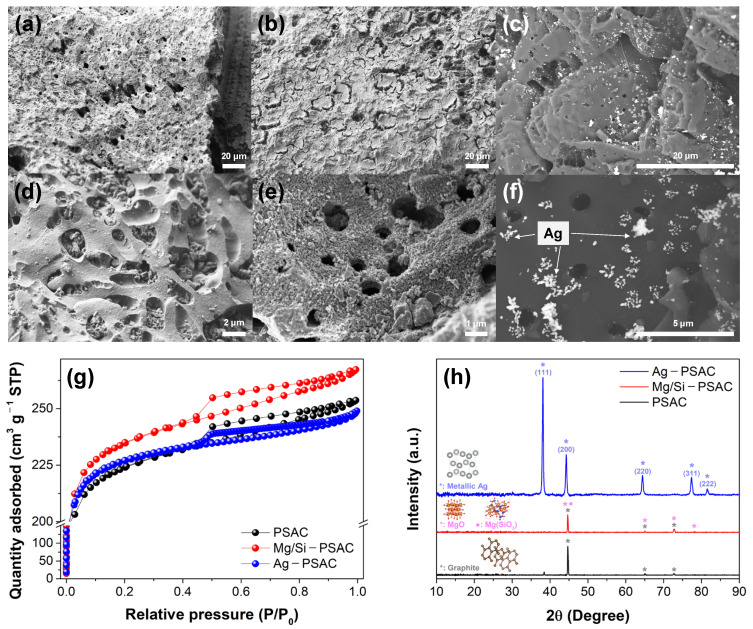
FESEM images of (**a**,**d**) PSAC, (**b**,**e**) Mg/Si-PSAC, and (**c**,**f**) Ag-PSAC (backscattered mode). (**g**) N_2_ adsorption-desorption isotherms for PSAC, Mg/Si-PSAC, and Ag-PSAC; (**h**) XRD spectra of PSAC, Mg/Si-PSAC, and Ag-PSAC.

The surface morphologies of PSAC, Mg/Si-PSAC, and Ag-PSAC were examined using FESEM-EDX (Figure 2a–c). PSAC and metal–PSAC exhibited honeycomb-like surface morphologies. The thin MgSiO_3_ layers and Ag nanoparticles on Mg/Si-PSAC and Ag-PSAC confirmed the successful anchoring of Mg/Si and Ag to the PSAC (Figure 2d–f), respectively. An N_2_ adsorption–desorption isotherm analysis was performed to investigate the pore structures of the media. The N_2_ adsorption–desorption isotherms (Figure 2g) and pore-size distributions (Figure S8) of PSAC, Mg/Si-PSAC, and Ag-PSAC revealed Type I isotherms and predominant micropore structures, according to the International Union of Pure and Applied Chemistry (IUPAC) classification [23]. This indicates that Mg/Si or Ag impregnation on the surface of the PSAC did not change its microporous structural properties. Table S7 summarizes the pore properties of PSAC, Mg/Si-PSAC, and Ag-PSAC. After Mg/Si modification, the specific surface area and total pore volume of the PSAC increased from 827.95 m^2^ g^−1^ and 0.392 cm^3^ g^−1^ to 887.22 m^2^ g^−1^ and 0.413 cm^3^ g^−1^, while the average pore size decreased from 1.896 to 1.864 nm. Mg/Si impregnation increased the PSAC surface area by forming thin MgSiO_3_ layers that caused additional pores. Meanwhile, Ag-PSAC also exhibited an increased specific surface area (887.34 m^2^ g^−1^) compared to PSAC, while the total pore volume (0.385 cm^3^ g^−1^) and average pore size (1.736 nm) were slightly decreased. This indicated that the Ag nanoparticles were either coated within the macropores or doped onto the surface of the PSAC. Neither the MgSiO_3_ layers nor Ag nanoparticles blocked the micropores, allowing Mg/Si-PSAC and Ag-PSAC to maintain their high surface areas. These additional active sites and increased surface area and provided by the MgSiO_3_ layers or Ag nanoparticles can enhance the heavy metal adsorption or disinfection capability of PSAC.

XRD analysis was performed to assess the crystallinity and structural changes in the adsorbents before and after surface modification (Figure 2e). The amorphous carbon peak at 20–30°, which is present in all three activated carbons [24], was omitted via baseline adjustment to clarify the other crystalline peaks. Graphite peaks were detected for PSAC at 45°, 65°, and 73° (JCPDS no. 75-2078) [25]. For Mg/Si-PSAC, the diffraction peaks at 45°, 65°, 73°, and 78° were identified as MgO (JCPDS no. 87-0653) [26], MgSiO_3_ (JCPDS no. 00-047-1750) [27], and graphite, respectively. Highly crystalline peaks of cubic metallic Ag at 38°, 44°, 64°, 78°, and 82° (JCPDS no. 04-0783), corresponding to the (1 1 1), (2 0 0), (2 2 0), (3 1 1), and (2 2 2) planes, respectively, were observed for Ag-PSAC, revealing the successful impregnation of Ag nanoparticles [28].

An FTIR analysis was conducted to investigate the surface functional groups of PSAC, Mg/Si-PSAC, and Ag-PSAC (Figure S8). The large particle size of granular carbon media makes them less suitable than powdered media for observing surface functional groups [29]. Thus, the PSACs were crushed to reduce their particle size prior to FTIR analysis. The typical activated carbon peaks for C-O (1115 cm^−1^), C=C in aromatic rings (1560 cm^−1^), C-H stretching (2980–2900 cm^−1^), and O-H stretching (3730 cm^−1^) were observed on all three PSACs [30,31]. No additional Mg- or Ag-related peaks were detected for Mg/Si-PSAC or Ag-PSAC after impregnation. Additional peaks at 1003 and 965 cm^−1^, corresponding to Si-O-Si/Si-O-Me and Si-OH, were observed for Mg/Si-PSAC, indicating the presence of SiO_2_ and MgSiO_3_ on the surface [32]. This silicate bonding may enhance heavy metal adsorption on Mg/Si-PSAC [13].

### 3.3. Investigation of Application Potential for MPECS

#### 3.3.1. *Escherichia coli* Removal

**Figure 3:**
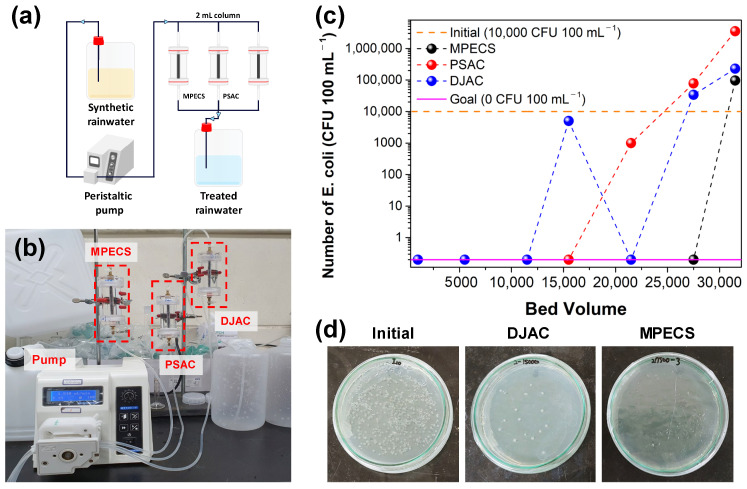
(**a**,**b**) Fixed-bed column experimental setup, (**c**) comparison column experiment results using MPECS, PSAC, and DJAC for E. coli removal, (**d**) *E. coli* colony photo for initial solution, DJAC at 15,000 BV, MPECS at 27,500 BV.

To meet drinking water guidelines in South Korea, *E. coli* must not be detected in water bodies. The MPECS column showed no *E. coli* detection until 27,500 BV (Figure 3, Table S8), but levels above the initial population (1.0 × 10^4^ CFU 100 mL^−1^) were detected after 31,500 BV. In contrast, *E. coli* was detected after 21,500 BV in the PSAC column and after 15,500 BV in the DJAC column, indicating an earlier breakthrough than in the MPECS column. Moreover, more *E. coli* exceeding the initial population was detected in PSAC and DJAC than in MPECS. This revealed that the MPECS had a better *E. coli* removal performance and inhibited microbial growth within the column media. The MPECS column demonstrated a 1.47 and 2.03 times greater *E. coli* removal capability than PSAC and DJAC, respectively. This result may be due to the Ag nanoparticles (shown in the FE-SEM) on Ag-PSAC in the MPECS column penetrating the *E. coli* cell walls, disrupting transport proteins, and causing cell starvation and death [14]. AgNPs also damage the cytoplasm of *E. coli*, thereby inhibiting their growth [27].

#### 3.3.2. Heavy Metal and Organic Pollutant Removal

**Figure 4:**
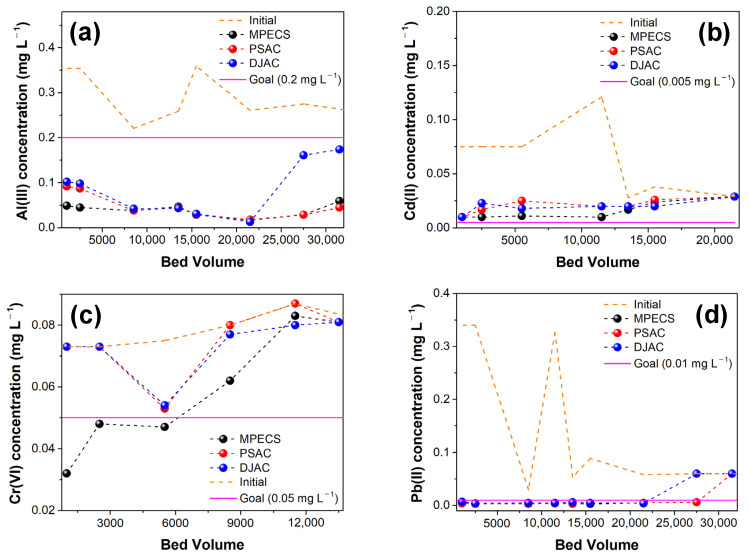
Comparative column experiment results using MPECS, PSAC, and DJAC columns for heavy metal removal: (**a**) Al(III), (**b**) Cd(II), (**c**) Cr(VI), and (**d**) Pb(II).

Synthetic rainwater with elevated levels of Al(III), Cd(II), Cr(VI), and Pb(II) was used to assess the breakthroughs and endpoints of the MPECS column for heavy metal removal. Unlike the batch experiments, Al(III) monitoring was also conducted for the synthetic rainwater, as rainwater in South Korea often contains high Al(III) concentrations because of springtime dust and aluminum gutters or pipes [18]. All three columns exhibited a strong Al(III) removal performance, sustaining up to 31,500 BV of Al(III) remediation (Figure 4a, Table S9). The Al(III) removal performance of the DJAC column declined after 21,500 BV, indicating that the PSAC-based media (PSAC and MPECS) were more effective for Al(III) adsorption than DJAC. This is due to the lower surface area and larger particle size of DJAC compared to PSAC, implying that DJAC has fewer adsorption sites for the removal of heavy metals such as Al(III). Cd(II) is generally undetectable or present in trace amounts in rainwater. However, to determine the breakthrough points, the initial Cd(II) concentration was set at 0.028–0.128 mg L^−1^, 5.6–25.6 times higher than Korea’s drinking water guideline (0.005 mg L^−1^). All columns reached the endpoint (C_e_/C_0_ = 1) after 21,500 BV, but the MPECS column exhibited superior overall Cd(II) removal compared to the control columns (PSAC or DJAC) (Figure 4b, Table S10). For Cr(VI) removal (influent concentration: 0.073–0.087 mg L^−1^), MPECS column breakthrough occurred at 5500 BV, with the endpoint at 13,500 BV (Figure 4c, Table S11). The PSAC and DJAC columns showed poor Cr(VI) removal performances compared to the MPECS. The MPECS column also exhibited strong Pb(II) adsorption, reaching the endpoint after 31,500 BV, similar to that of the PSAC (Figure 4d, Table S12). DJAC reached the endpoint earlier, at 27,500 BV. Overall, consistent with the batch results (Figure 1), Mg/Si and Ag impregnation improved the Cd(II) removal efficiency of the PSAC, whereas Mg/Si-PSAC in the MPECS provided additional sorption sites for Cr(VI). The PSAC-based media also demonstrated high Pb(II) adsorption.

Moreover, the removal performance of MPECS for organic matter was investigated by measuring the chemical oxygen demand (COD) (Figure S9, Table S13). All three columns effectively reduced COD concentrations. MPECS showed superior performance, reducing the COD to below 12 mg L^−1^ after 1000 BV, meeting Korea’s discharge water quality guidelines [33]. Mg/Si-PSAC and Ag-PSAC in the MPECS column enhanced the removal of heavy metals and COD by providing additional active sites. MPECS reduced *E. coli* concentrations to meet no-detection drinking water guidelines by up to 31,500 BV.

In practical use, assuming 30,000 BV with a safety factor, a 10 L MPECS column can treat up to 300,000 L (300 m^3^) of rainwater. A 10 L MPECS column can remove *E. coli* for one year, assuming a 300 m^2^ catchment area and 1000 mm annual rainfall. The removal capacity of the MPECS can be adjusted by modifying the media volume to suit the contamination level, making it an efficient multi-pollutant treatment solution.

## 4. Discussion

**Figure 5:**
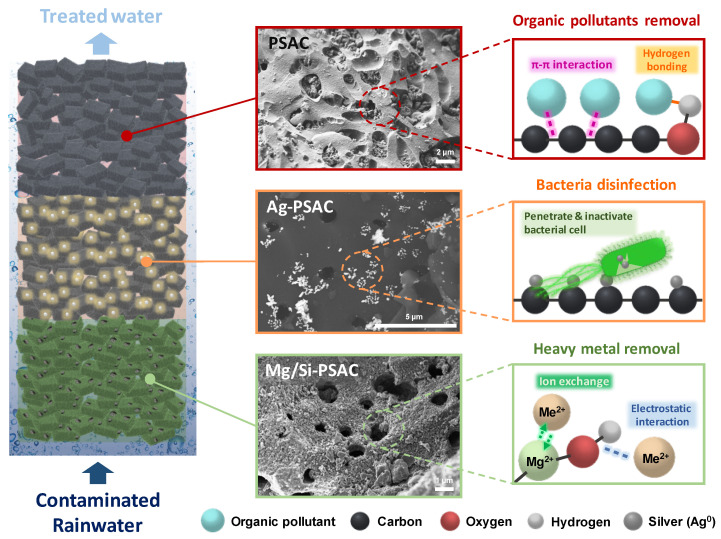
Proposed mechanism for heavy metals, organic pollutants, and *E. coli* removal using PSAC, Mg/Si-PSAC, and Ag-PSAC in MPECS column.

The MPECS medium effectively removed *E. coli*, heavy metals, and COD from water resources such as rainwater. PSAC, Mg/Si-PSAC, and Ag-PSAC in an MPECS remove pollutants through distinct mechanisms owing to their varied surface functional groups (Figure 5). PSAC’s surface functional groups, including O-H, C-H, C=C, and C=O bonds, have a strong affinity for removing natural organic matter [34]. The C=C bond in PSAC’s aromatic rings removes aromatic compounds through π-π interactions, while the O-H groups form hydrogen bonds with organic pollutants [35]. The π-π interactions are known as π-π electron donor–acceptor interactions, which are between the π-electrons in a carbon-based adsorbent and the π-electron in the aromatic ring of an adsorbate [36]. Moreover, the oxygen functional groups, such as C-OH or -COOH bonds, on the PSAC surface can act as electron donors, while the aromatic rings of organic pollutants act as electron acceptors (n-π interaction) (Equation (1)) [37]. Furthermore, the H groups of PSACs can remove heavy metals (designated Me^+^) via electrostatic interactions (Equation (2)).

PSAC-OH + Organic pollutant ↔ PSAC-O ⋯ Organic pollutant(1)

PSAC-OH + Me^+^ ↔ PSAC-O^−^ ⋯ Me^+^ (2)

Mg/Si-PSAC has a higher specific surface area (887.22 m^2^ g^−1^) than PSAC due to thin MgSiO_3_ layers and additional pores, which enhance meso- and macropore structures without clogging the PSAC’s internal pores. Mg/Si-PSAC has surface functional groups, such as O-H, C-H, C=C, and C=O, and an additional Mg-OH bond from Mg/Si impregnation. The Mg(II) in the Mg-OH groups bond with oxygen from the silicate groups, forming C-O-Si-O-Mg-OH on the PSAC surface. Mg-OH exhibits a high selectivity for heavy metal adsorption via electrostatic interactions and ion exchange [38]. In batch experiments (Figure 1), Mg/Si-PSAC showed a superior adsorption of Cd(II), Cr(VI), and Pb(II) in both single- and multi-metal systems. For Cd(II) and Pb(II) removal, the Mg(II) in Mg-OH on Mg/Si-PSAC is exchanged with Cd(II) or Pb(II), forming Cd-OH or Pb-OH bonds (Equation (3)). Moreover, the Mg-OH bonded with Si-O can exchange with metals, forming Si-O-Me^⁺^ (Equation (4)) [25].

PSAC-Si-O-Mg-OH + Me(II) ↔ PSAC-Si-O-Me-OH + Mg(II)(3)

PSAC-Si-O-Mg-OH + Me(II) ↔ PSAC-Si-O-Me^+^ + Mg(II) + OH^−^ (4)

In the batch experiments, the amount of Pb(II) adsorbed on Mg/Si-PSAC was significantly higher than that adsorbed on Cd(II) in the presence and absence of *E. coli*. The smaller size of the hydrated Pb(II) ions compared to Cd(II) ions can inhibit the steric hindrance effect towards interaction with adsorbents, leading to better adsorption efficiencies [39]. Due to its higher electronegativity (1.80–2.33) compared to Cd(II) (1.69–1.70), Pb(II) has a greater affinity to remaining adsorbed on Mg/Si-PSAC [40]. Additionally, the Mg-OH groups on Mg/Si-PSAC, a hard Lewis base, strongly interact with Pb(II) ions, which are hard Lewis acids [38]. However, as soft Lewis acids, Cd(II) ions exhibit weak interactions, resulting in a relatively low Mg/Si-PSAC removal efficiency [41]. Unlike Cd(II) and Pb(II), Cr(VI) exists as negative charged species like HCrO_4_^−^ (pH 1–6), Cr_2_O_7_^2−^ (pH 2–6), and CrO_4_^2−^ (pH ≥ 7) in an aqueous solution [42]. Thus, Cr(VI) is directly adsorbed onto the positively charged magnesium silicate groups of Mg/Si-PSAC (Equation (5)).

PSAC-Si-O-Mg-OH + Cr(VI) ↔ PSAC-Si-O-Mg-OH_2_^+^-Cr(VI) + OH^−^ (5)

Ag-PSAC, with Ag nanoparticles embedded in PSAC, removes *E. coli* through a combination of adsorption and disinfection. *E. coli* is first adsorbed onto Ag-PSAC via hydrophobic and electrostatic interactions [43]. After adsorption, the Ag nanoparticles on the Ag-PSAC began to disinfect the bacteria. AgNPs penetrate the bacterial cell walls through ion channels, inactivate proteins, damage the cell wall, and disinfect the *E. coli*. Additionally, AgNPs disrupts *E. coli* growth by damaging the electron-dense cytoplasm [16]. Meanwhile, Ag-PSAC showed better Cd(II) and Pb(II) removal than PSAC because Ag nanoparticles act as coagulants, promoting heavy metal precipitation [44,45].

## 5. Conclusions

This study is the first to explore the potential of MPECS for the removal of multiple pollutants (heavy metals, COD, and *E. coli*) from rainwater. Mg/Si- and Ag-impregnated PSAC showed superior heavy metal removal compared to PSAC, even in the presence of multiple heavy metals and *E. coli*. The combination of PSAC, Mg/Si-PSAC, and Ag-PSAC further improved the heavy metal removal, even in the presence of multiple heavy metals and *E. coli*. Mg/Si and Ag impregnation on PSAC added active sites for heavy metal adsorption and microorganism disinfection without blocking the pores. Comparative experiments showed that the MPECS column excelled in simultaneously removing heavy metals, COD, and *E. coli*. MPECS demonstrated superior *E. coli* disinfection, with a 1.47 and 2.03 times greater removal than PSAC and DJAC, respectively. The MPECS column inhibited *E. coli* growth up to 31,500 BV, meeting drinking water guidelines (N.D.). The MPECS column showed superior heavy metal removal compared to the control columns, owing to the presence of Mg/Si-PSAC in its media. The Mg-OH in the Mg/Si-PSAC has a high selectivity for adsorbing and exchanging heavy metals, such as Cd(II), Cr(VI), and Pb(II). The aromatic C=C groups of PSAC favor organic matter adsorption, whereas the O-H groups remove heavy metals such as Pb(II) through electrostatic interactions. MPECS is a promising solution for treating multiple pollutants in water sources such as rainwater.

## Figures and Tables

**Table 1:** Event mean concentrations (EMCs) for heavy metals and E. coli of rainwater at monitoring sites in South Korea reported in previous studies.

Monitoring	Cr (µg L^−1^)	Cu (µg L^−1^)	Cd (µg L^−1^)	Pb (µg L^−1^)	*E. coli* (MPN 100 mL^−1^)	Ref.
22 June 2011	10.27	12.73	0.74	7.51	ND	[17]
7 July 2011	15.83	7.62	0.03	4.34	ND	
26 July 2011	21.44	17.19	0.15	12.07	ND	
3 August 2011	37.76	11.40	0.00	11.81	ND	
12 August 2011	3.28	7.91	0.68	21.63	ND	
29 September 2011	ND	ND	ND	ND	ND	
Mean	17.72	11.37	0.32	11.47	ND	
Minimum	3.28	7.62	0.00	4.34	ND	
Maximum	37.76	17.19	0.74	21.63	ND	
Rainwater in storage tank	-	30–70	-	-	0–21	[19]
Roof runoff	-	20–50	-	-	ND	
Roof garden runoff	-	30–360	-	-	ND	

## Data Availability

The data supporting the findings of this study are available upon request from the corresponding author.

## Supplementary Materials

The following supporting information can be downloaded at: https://www.mdpi.com/article/10.3390/ma17225638/s1, Figure S1: (a) Magnesium-modified PSAC (Mg-PSAC) and (b) silver-modified PSAC (Ag-PSAC); Figure S2: The composition of MPECS filter for fixed-bed column experiment; Figure S3: Heavy metal removal using PSAC, Mg/Si-PSAC, and Ag-PSAC in the presence of single heavy metals [Cd(II), Cr(VI), and Pb(II)]; Figure S4: Heavy metal removal using PSAC, Mg/Si-PSAC, and Ag-PSAC in the presence of Cd(II), Cr(VI), and Pb(II) together; Figure S5: Heavy metal removal using PSAC, Mg/Si-PSAC, and Ag-PSAC in the presence of Cd(II), Cr(VI), Pb(II), and *E. coli* together; Figure S6: Heavy metal removal using different carbon media combinations in the presence of Cd(II), Cr(VI), Pb(II), and *E. coli* together; Figure S7: The pore-size distribution of (a) PSAC, Mg/Si-PSAC, and (b) Ag-PSAC; Figure S8: FTIR spectra of PSAC, Mg/Si-PSAC, and Ag-PSAC; Figure S9: Comparison column experiment results using MPECS, PSAC, and DJAC for COD removal; Table S1: Detailed configuration of fixed-bed column experiments; Table S2: The heavy metal removal efficiencies of PSAC, Mg/Si-PSAC, and Ag-PSAC in the presence of a single metal; Table S3: The parameters of pseudo-first- and second-order kinetic models for Cd(II), Cr(VI), and Pb(II) adsorption onto PSAC, Mg/Si-PSAC, and Ag-PSAC; Table S4: The heavy metal removal efficiencies of PSAC, Mg/Si-PSAC, and Ag-PSAC in the presence of multi-metal; Table S5: The heavy metal removal efficiencies of PSAC, Mg/Si-PSAC, and Ag-PSAC in the presence of multi-metal and *E. coli*; Table S6: The heavy metal removal efficiencies of PSAC, Mg/Si-PSAC, and Ag-PSAC in the presence of multi-metal and E. coli; Table S7. Surface area, total pore volume, average pore size, and micro-pore volume of PSAC and Mg/Si-PSAC; Table S8: Comparison fixed-bed column experiment results of MPECS, PSAC, and DJAC column for E. coli removal; Table S9: Comparison fixed-bed column experiment results of MPECS, PSAC, and DJAC column for Al(III) removal; Table S10: Comparison fixed-bed column experiment results of MPECS, PSAC, and DJAC column for Cd(II) removal; Table S11: Comparison fixed-bed column experiment results of MPECS, PSAC, and DJAC column for Cr(VI) removal; Table S12: Comparison fixed-bed column experiment results of MPECS, PSAC, and DJAC column for Pb(II) removal; Table S13: Comparison fixed-bed column experiment results of MPECS, PSAC, and DJAC column for COD removal.
