# Supplementary material for: Mg/Si- and Ag-Doped Carbon-Based Media Rainwater Filtration System for Multiple Pollutants Removal
Source: Materials (Basel). 2024 Nov 18;17(22):5638. doi: 10.3390/ma17225638 (PMC11595580; doi:10.3390/ma17225638)
Supplement: Supplementary file 1 [file materials-17-05638-s001.zip › materials-3310571-supplementary.pdf]

Supplementary information

Mg/Si and Ag-doped carbon based media rainwater filtration system for multiple pollutants removal

So Yeon Yoon[a,b†], Hyeseong Kim[a†], Reneesha Valiyaveettil Basheer[a], Nurhaslina Abd Rahman[a,b], Seok Byum Jang[a], Kien Tiek Wong[a,b], Deok Hyun Moon [c], Choe Earn Choong[a,b*] and Min Jang[a,b**]

[a]Department of Environmental Engineering, Kwangwoon University, 20 Kwangwoon-Ro, Nowon-Gu, Seoul 01897, Republic of Korea

[b]Plasma Bioscience Research Center, Dasanjae 101, Kwangwoon University, 20 Kwangwoon-Ro, Nowon-Gu, Seoul 01897, Republic of Korea

[c]Department of Environmental Engineering, Chosun University, Gwangju 61452, Republic of Korea

[*]Corresponding author. Email: cce_@live.com;

[**]Co-corresponding author. Email: minjang@kw.ac.kr (heejaejang@gmail.com); tel: (+82) 2-940-5125

[†]These authors contributed equally as co-first authors to this work.

S-1: Materials

Sliver nitrate ($AgNO_3$), sodium borohydride ($NaBH_4$), silicon dioxide (SiO_2), magnesium oxide (MgO), sodium hydroxide (NaOH), potassium dichromate ($K_2Cr_2O_7$), cadmium nitrate tetrahydrate ($Cd(NO_3)_2 \cdot 4H_2O$), aluminum nitrate nonahydrate ($Al(NO_3)_3 \cdot 9H_2O$), lead nitrate ($Pb(NO_3)_2$), potassium phosphate monobasic (KH_2PO_4), potassium persulfate ($K_2S_2O_8$), potassium nitrate (KNO_3), and granular activated carbon (mesh size 4x8, specific surface area: ≥900 m^2 g, named as DJAC) were obtained from Daejung Chemicals & Metals Co., Ltd, South Korea. All the chemicals were used in experiments without further treatment. Ultrapure water (18.2 MΩ-cm) was used for preparing all solutions for batch and column experiments. The palm shell waste based granular activated carbon (PSAC) was obtained from Bravo Green Sdn. Bhd, Malaysia pass through mesh size 20x40, iodine number: 900-1050 mg g^{-1}, moisture content: 5%, ash content: 5%, bulk density: 0.40-0.46 g mL^{-1}.Before use for experiments, PSAC was washed with distilled water more than 3 times until washed water electro-conductivity was less than 300 μs cm^{-1} and oven dried at 60°C for 24 h.

S-2: Kinetic models

The single heavy metals [Cd(II), Cr(VI), and Pb(II)] kinetic adsorption data were fitted to pseudo-first-order and pseudo-second-order kinetic models. The following equation gives the equation of the pseudo-first-order kinetic model.

$$\ln(q_e - q_t) = \ln q_e - k_1 t \tag{4}$$

Where q_e (mg g^{-1}) and q_t (mg g^{-1}) are the amounts of adsorbed heavy metals by an adsorbent at equilibrium and at time t (min). The k_1 (min^{-1}) is the rate constant of the pseudo-first-order reaction.

The following equation gives the equation of the pseudo-second-order kinetic model:

$$\frac{t}{q_t} = \frac{1}{k_2 q_e^2} + \frac{t}{q_e} \tag{5}$$

where q_e (mg g^{-1}) and q_t (mg g^{-1}) are the adsorbed amounts of heavy metals at equilibrium and at time t (min), and k_2 (min^{-1}) is the rate constant of the pseudo-second-order reaction.

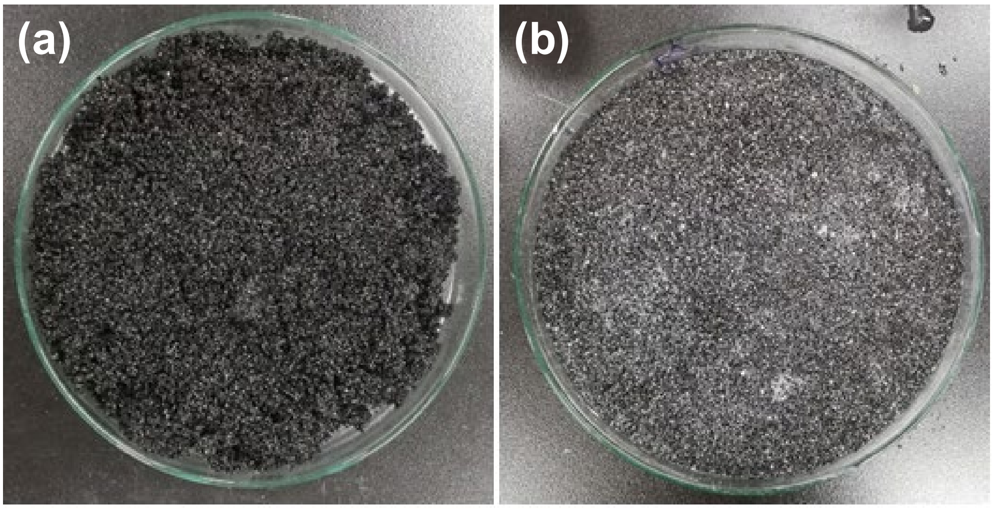

Figure S1. (a) Magnesium modified PSAC (Mg-PSAC) and (b) silver modified PSAC (Ag-PSAC).

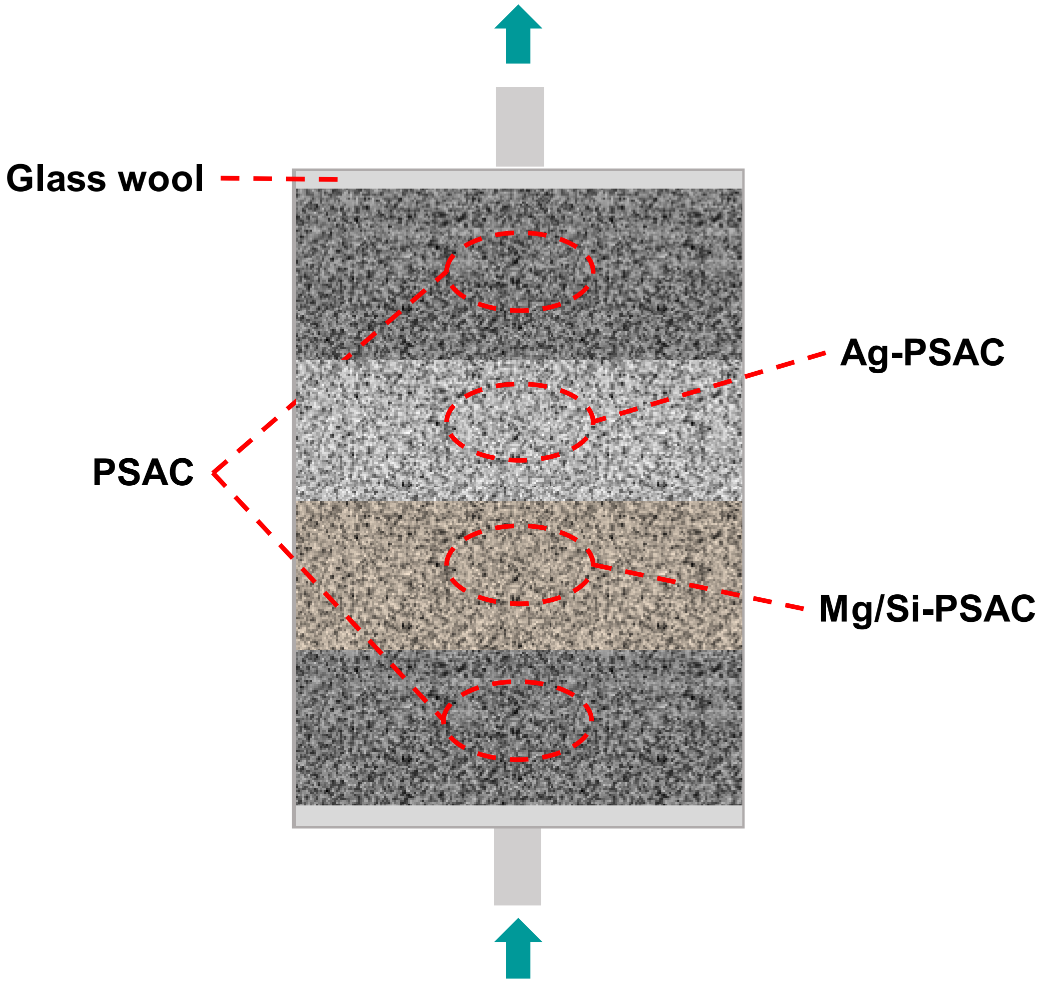

Figure S2. The composition of MPECS filter for fixed-bed column experiment.

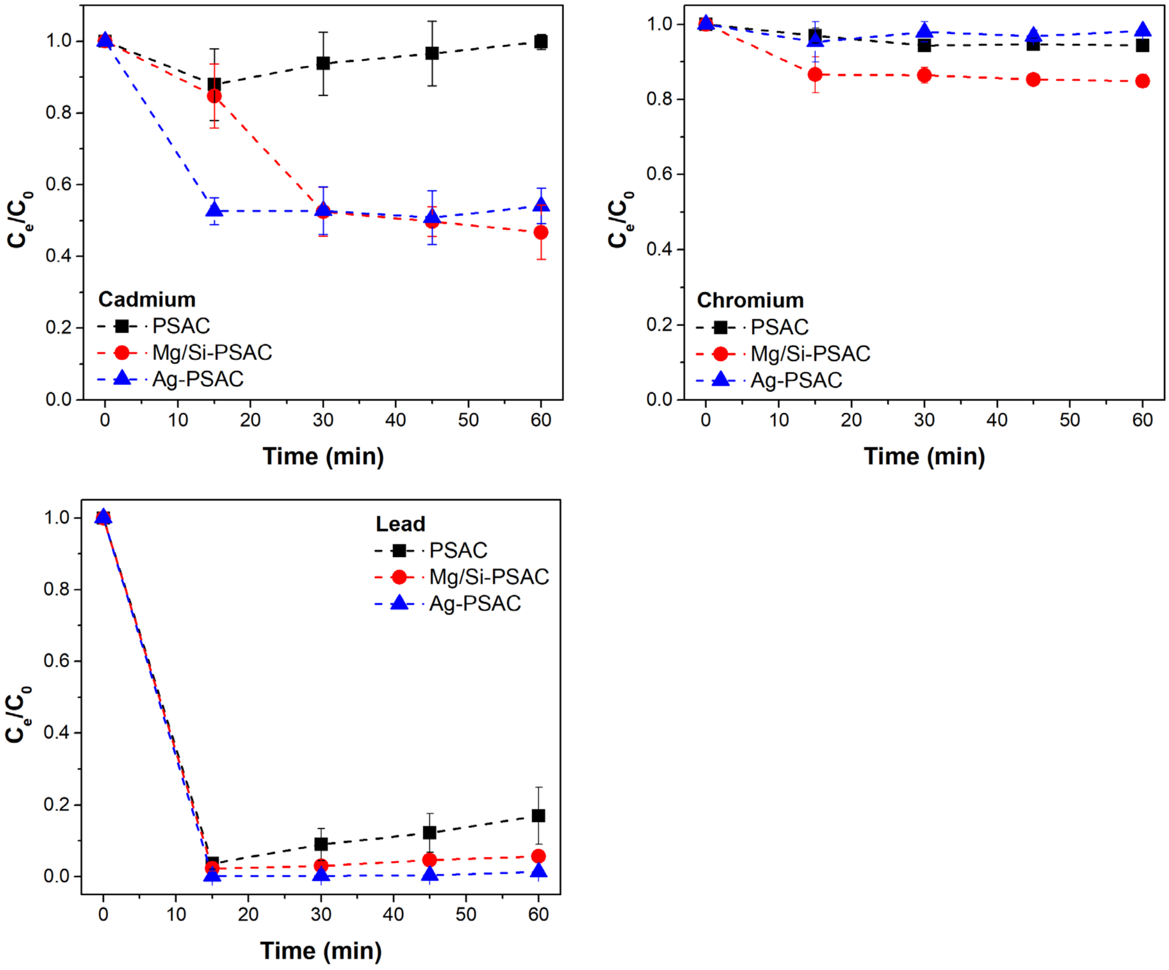

Figure S3. Heavy metal removal using PSAC, Mg/Si-PSAC, and Ag-PSAC in the presence of single heavy metals (Cd(II), Cr(VI), and Pb(II)) (Initial heavy metal concentration: 1 mg L^{-1}, dosage: 0.1 g, solution volume: 200 mL, RT).

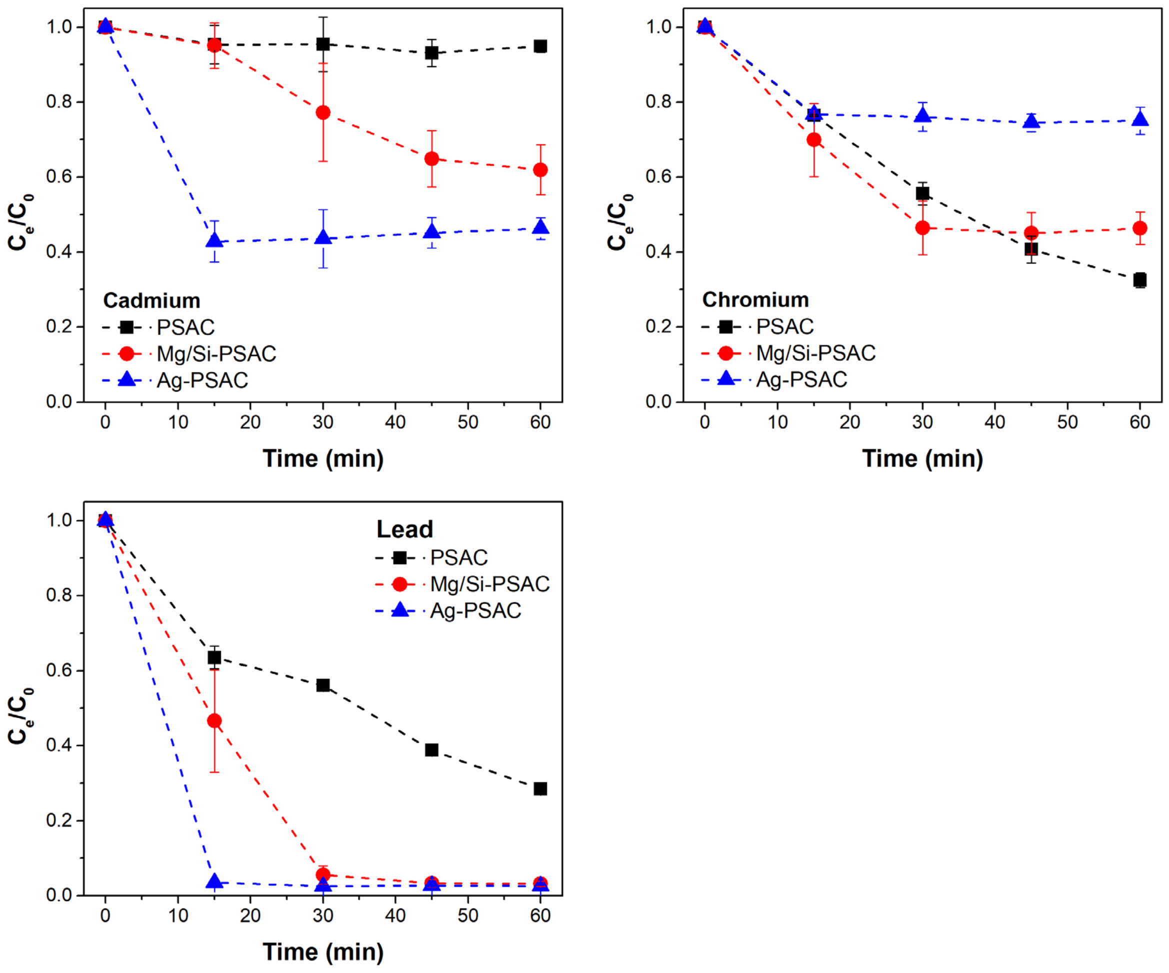

Figure S4. Heavy metal removal using PSAC, Mg/Si-PSAC, and Ag-PSAC in the presence of Cd(II), Cr(VI), and Pb(II) together (Initial heavy metal concentration: 1 mg L^{-1}, dosage: 0.1 g, solution volume: 200 mL, RT).

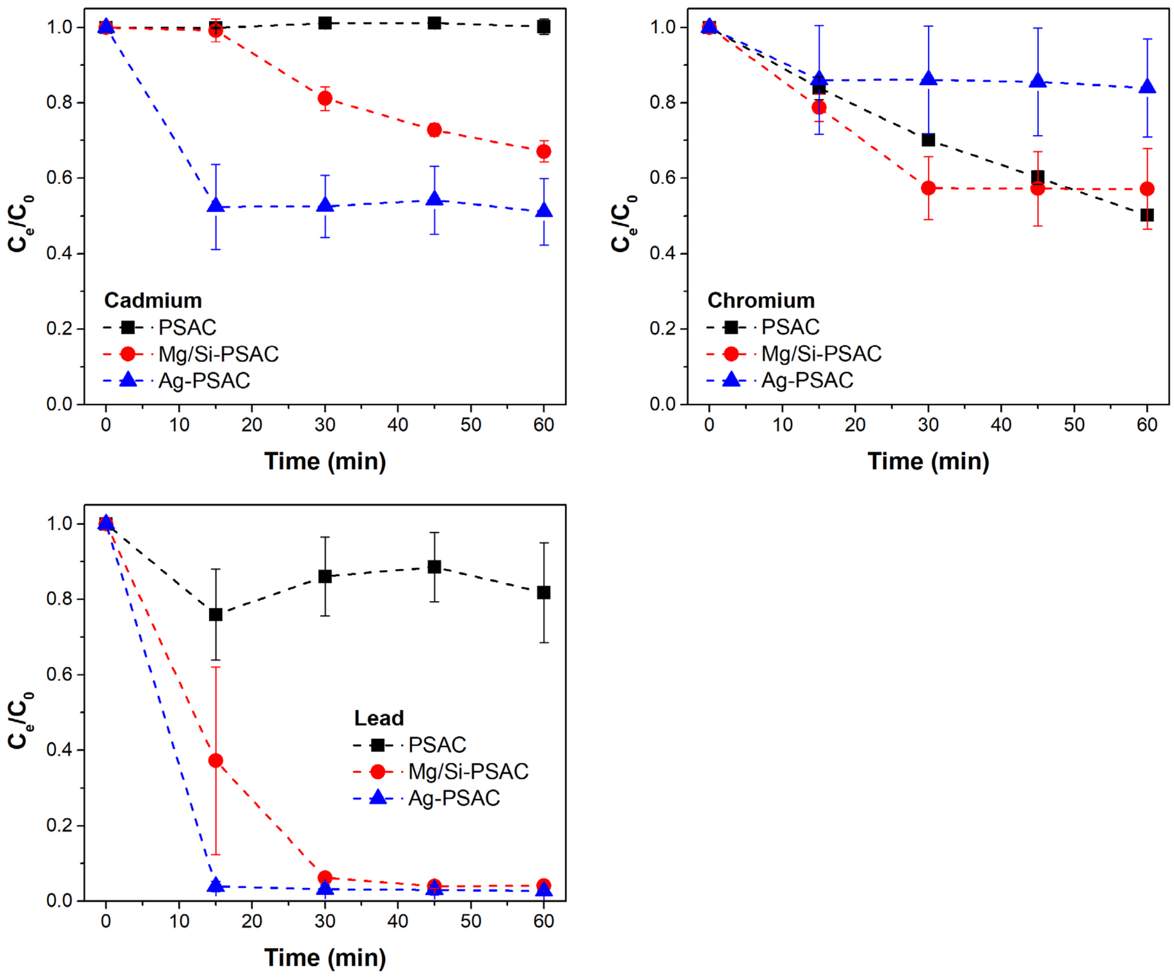

Figure S5. Heavy metal removal using PSAC, Mg/Si-PSAC, and Ag-PSAC in the presence of Cd(II), Cr(VI), Pb(II), and E. coli together (Initial heavy metal concentration: 1 mg L^{-1}, initial E. coli population: 1.0 x 10^4 CFU 100 mL^{-1}, dosage: 0.1 g, solution volume: 200 mL, RT).

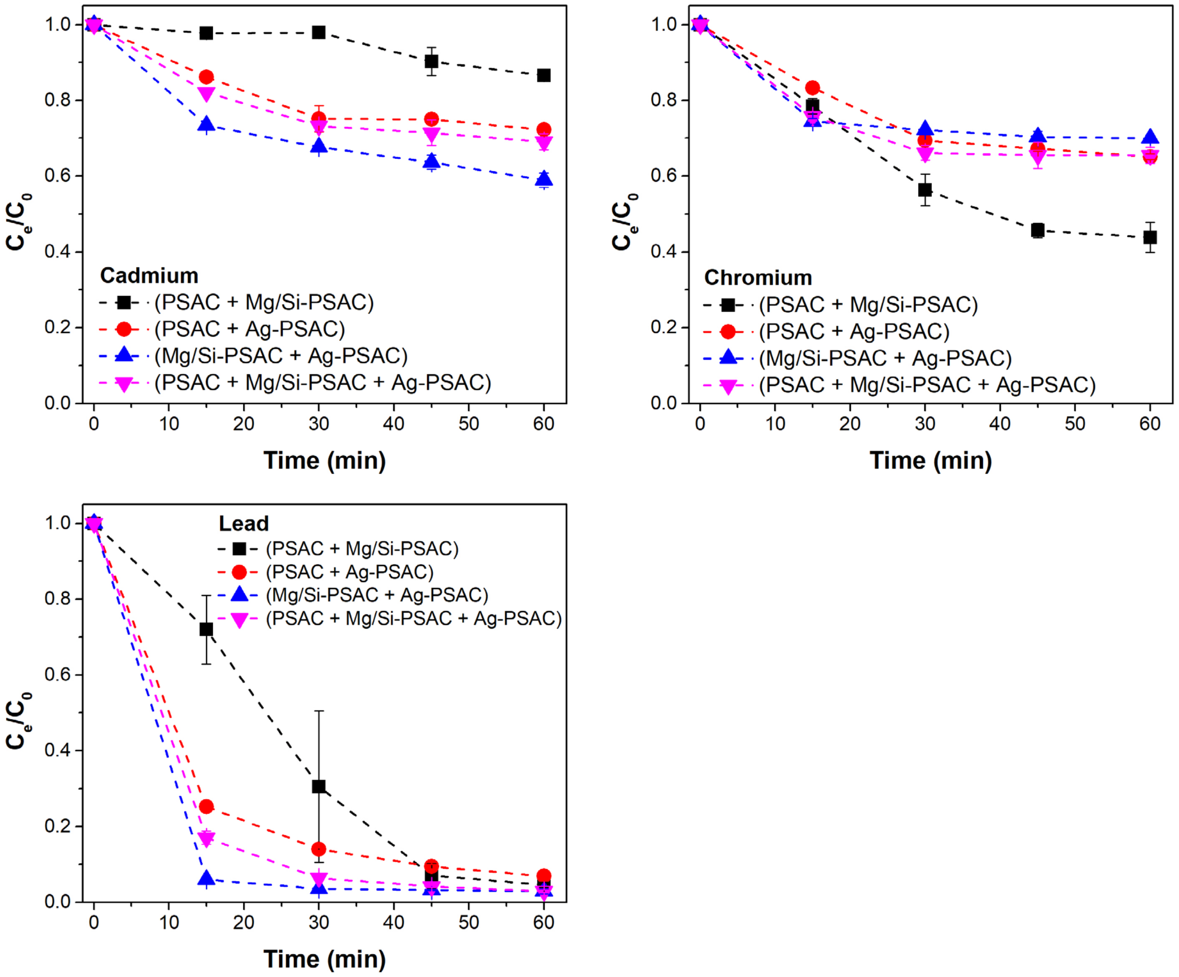

Figure S6. Heavy metal removal using different carbon media combinations in the presence of Cd(II), Cr(VI), Pb(II), and E. coli together (combination 1 – PSAC + Mg/Si-PSAC, 2 – PSAC + Ag-PSAC, 3 – Mg/Si-PSAC + Ag-PSAC, 4 – PSAC + Mg/Si-PSAC + Ag-PSAC) (Initial heavy metal concentration: 1 mg L^{-1}, initial E. coli population: 1.0 x 10^4 CFU 100 mL^{-1}, dosage: 0.1 g, solution volume: 200 mL, RT).

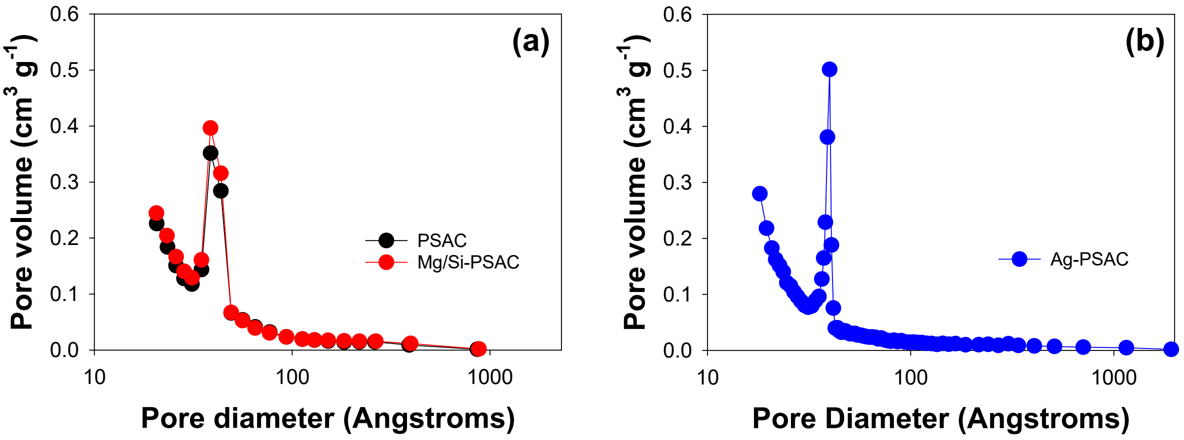

Figure S7. The pore size distribution of (a) PSAC, Mg/Si-PSAC, and (b) Ag-PSAC.

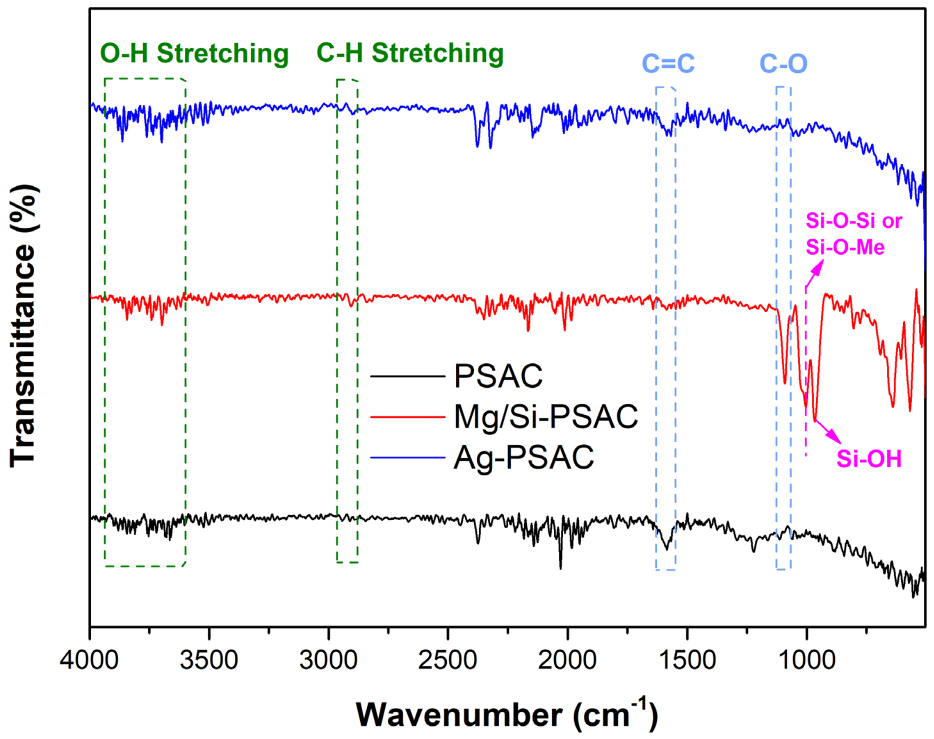

Figure S8. FTIR spectra of PSAC, Mg/Si-PSAC, and Ag-PSAC.

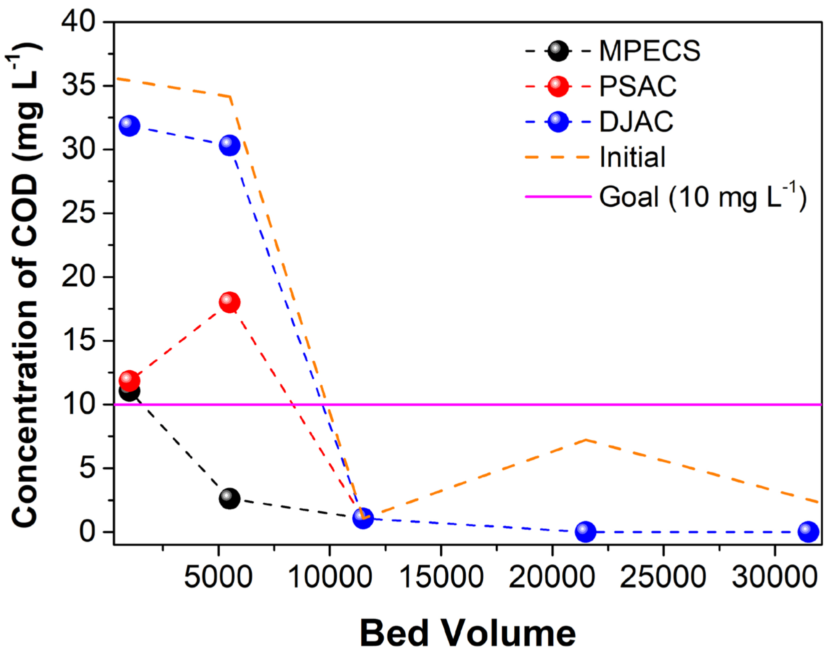

Figure S9. Comparison column experiments results using MPECS, PSAC, and DJAC for COD removal.

Table S1. The detail configuration of fixed-bed column experiments.

Column parameter	Value	Unit
**Column height**	10	cm
**Column diameter**	Outer: 4<br>Inner: 0.6	cm
**Flow rate**	4	mL min^{-1}
**Bed volume (BV)**	2.0	mL
**EBCT**	0.5	min

Table S2. The heavy metal removal efficiencies of PSAC, Mg/Si-PSAC, and Ag-PSAC in the presence of single metal.

Adsorbents	Cd(II) removal efficiency (%)	Cr(VI) removal efficiency (%)	Pb(II) removal efficiency (%)
PSAC	0.15	5.66	83.05
Mg/Si-PSAC	53.24	15.13	94.36
Ag-PSAC	45.95	1.72	98.65

Table S3. The parameters of pseudo first and second-order kinetic models for Cd(II), Cr(VI), and Pb(II) adsorption onto PSAC, Mg/Si-PSAC, and Ag-PSAC.

Models	Pseudo first-order model		Pseudo second-order model	
Adsorbents	k_1 (min^{-1})	R^2	k_2 (g mg^{-1} min^{-1})	R^2
**PSAC [Cd(II)]**	0.004	0.9988	0.004	0.9495
**Mg/Si-PSAC [Cd(II)]**	0.005	0.8683	0.002	0.1866
**Ag-PSAC [Cd(II)]**	0.050	0.8389	0.387	0.9997
**PSAC [Cr(VI)]**	0.024	0.7807	0.405	0.9302
**Mg/Si-PSAC [Cr(VI)]**	0.044	0.8518	0.833	0.9972
**Ag-PSAC [Cr(VI)]**	0.007	0.9549	0.027	0.2778
**PSAC [Pb(II)]**	0.044	0.8698	0.121	0.9974
**Mg/Si-PSAC [Pb(II)]**	0.063	0.7739	0.552	0.9999
**Ag-PSAC [Pb(II)]**	0.082	0.7069	2.324	0.9999

Table S4. The heavy metal removal efficiencies of PSAC, Mg/Si-PSAC, and Ag-PSAC in the presence of multi-metal.

Adsorbents	Cd(II) removal efficiency (%)	Cr(VI) removal efficiency (%)	Pb(II) removal efficiency (%)
PSAC	5.07	67.50	71.60
Mg/Si-PSAC	38.07	53.65	96.82
Ag-PSAC	53.73	24.98	97.39

Table S5. The heavy metal removal efficiencies of PSAC, Mg/Si-PSAC, and Ag-PSAC in the presence of multi-metal and e.coli.

Adsorbents	Cd(II) removal efficiency (%)	Cr(VI) removal efficiency (%)	Pb(II) removal efficiency (%)
PSAC	0	49.81	18.22
Mg/Si-PSAC	32.88	42.88	95.86
Ag-PSAC	48.89	16.04	97.27

Table S6. The heavy metal removal efficiencies of PSAC, Mg/Si-PSAC, and Ag-PSAC in the presence of multi-metal and e.coli.

Media combination	Cd(II) removal efficiency (%)	Cr(VI) removal efficiency (%)	Pb(II) removal efficiency (%)
1 (PSAC + Mg/Si-PSAC)	13.47	56.19	95.34
2 (PSAC + Ag-PSAC)	27.78	34.89	93.06
3 (Mg/Si-PSAC + Ag-PSAC)	41.05	30.06	97.07
4 (PSAC + Mg/Si-PSAC + Ag-PSAC)	30.91	34.53	97.03

Table S7. Surface area, total pore volume, average pore size and micro-pore volume of PSAC and Mg/Si-PSAC.

Adsorbent	Specific surface area (m^2 g^{-1})	Total Pore volume (cm^3 g^{-1})	Average Pore size (nm)
**PSAC**	827.95	0.392	1.896
**Mg/Si-PSAC**	887.22	0.413	1.864
**Ag-PSAC**	887.34	0.385	1.736

Table S8. Comparison fixed-bed column experiment results of MPECS, PSAC, and DJAC column for e.coli removal (bacteria population unit: CFU 100 mL^{-1}).

Bed volume (BV)	Initial	MPECS	PSAC	DJAC	Goal
**1000**		N.D.	N.D.	N.D.	
**5500**		N.D.	N.D.	N.D.	
**11500**		N.D.	N.D.	N.D.	
**15500**	1.0 x 10^4	N.D.	N.D.	5.0 x 10^3	N.D.
**21500**		N.D.	1.0 x 10^3	N.D.	
**27500**		N.D.	7.8 x 10^4	3.4 x 10^4	
**31500**		9.6 x 10^4	3.5 x 10^6	2.3 x 10^5	

Table S9. Comparison fixed-bed column experiment results of MPECS, PSAC, and DJAC column for Al(III) removal (unit: mg L^{-1}).

Bed volume (BV)	Initial	MPECS	PSAC	DJAC	Goal
**1000**	0.354	0.049	0.092	0.102	0.2
**2500**	0.354	0.045	0.087	0.098	
**8500**	0.221	0.038	0.038	0.042	
**13500**	0.259	0.047	0.045	0.043	
**15500**	0.359	0.029	0.030	0.031	
**21500**	0.261	0.017	0.018	0.013	
**27500**	0.275	0.029	0.029	0.161	
**31500**	0.254	0.059	0.045	0.174	

Table S10. Comparison fixed-bed column experiment results of MPECS, PSAC, and DJAC column for Cd(II) removal (unit: mg L^{-1}).

Bed volume (BV)	Initial	MPECS	PSAC	DJAC	Goal
**1000**	0.075	0.010	0.010	0.010	0.005
**2500**	0.075	0.010	0.017	0.023	
**5500**	0.075	0.011	0.025	0.018	
**11500**	0.121	0.010	0.020	0.020	
**13500**	0.028	0.017	0.020	0.020	
**15500**	0.038	0.024	0.026	0.020	
**21500**	0.029	0.029	0.029	0.029	

Table S11. Comparison fixed-bed column experiment results of MPECS, PSAC, and DJAC column for Cr(VI) removal (unit: mg L^{-1}).

Bed volume (BV)	Initial	MPECS	PSAC	DJAC	Goal
**1000**	0.073	0.032	0.073	0.073	0.005
**2500**	0.073	0.048	0.073	0.073	
**5500**	0.075	0.047	0.053	0.075	
**8500**	0.080	0.062	0.080	0.080	
**11500**	0.087	0.083	0.087	0.087	
**13500**	0.081	0.081	0.081	0.081	

Table S12. Comparison fixed-bed column experiment results of MPECS, PSAC, and DJAC column for Pb(II) removal (unit: mg L^{-1}).

Bed volume (BV)	Initial	MPECS	PSAC	DJAC	Goal
**1000**	0.340	0.004	0.004	0.007	0.010
**2500**	0.340	0.003	0.003	0.004	
**8500**	0.030	0.005	0.003	0.005	
**11500**	0.327	0.005	0.004	0.005	
**13500**	0.054	0.003	0.003	0.006	
**15500**	0.089	0.003	0.005	0.005	
**21500**	0.058	0.005	0.005	0.004	
**27500**	0.060	0.006	0.006	0.060	
**31500**	0.060	0.060	0.060	0.060	

Table S13. Comparison fixed-bed column experiment results of MPECS, PSAC, and DJAC column for COD removal (unit: mg L^{-1}).

Bed volume (BV)	Initial	MPECS	PSAC	DJAC	Goal
**1000**	35.7	11.1	11.8	31.8	10.0
**5500**	34.2	2.6	18.0	30.3	
**11500**	1.1	1.1	1.1	1.1	
**21500**	7.2	N.D.	N.D.	N.D.	
**31500**	1.8	N.D.	N.D.	N.D.	
